# New Perspective on Wood Thermal Modification: Relevance between the Evolution of Chemical Structure and Physical-Mechanical Properties, and Online Analysis of Release of VOCs

**DOI:** 10.3390/polym11071145

**Published:** 2019-07-04

**Authors:** Jiajia Xu, Yu Zhang, Yunfang Shen, Cong Li, Yanwei Wang, Zhongqing Ma, Weisheng Sun

**Affiliations:** 1 School of Engineering, Zhejiang Provincial Collaborative Innovation Center for Bamboo Resources and High-Efficiency Utilization, Zhejiang A & F University, Hangzhou 311300, Zhejiang, China; 2 Zhejiang Shenghua Yunfeng Greeneo Co. Ltd., Huzhou 313220, Zhejiang, China; 3 Treessun Flooring Co. Ltd., Huzhou 313009, Zhejiang, China

**Keywords:** wood, thermal modification, mechanical properties, dimensional stability, color, chemical structure, VOCs

## Abstract

Thermal modification (TM) is an ecological and low-cost pretreated method to improve the dimensional stability and decay resistance of wood. This study systematically investigates the relevance between the evolution of chemical structure and the physical and mechanical properties during wood thermal modification processes. Moreover, the volatility of compounds (VOCs) was analyzed using a thermogravimetric analyzer coupled with Fourier transform infrared spectrometry (TGA-FTIR) and a pyrolizer coupled with gas chromatography/mass spectrometer (Py-GC/MS). With an increase of TM temperature, the anti-shrink efficiency and contact angle increased, while the equilibrium moisture content decreased. This result indicates that the dimensional stability improved markedly due to the reduction of hydrophilic hydroxyl (–OH). However, a slight decrease of the moduli of elasticity and of rupture was observed after TM due to the thermal degradation of hemicellulose and cellulose. Based on a TGA-FTIR analysis, the small molecular gaseous components were composed of H_2_O, CH_4_, CO_2_, and CO, where H_2_O was the dominant component with the highest absorbance intensity, i.e., 0.008 at 200 °C. Based on the Py-GC/MS analysis, the VOCs were shown to be mainly composed of acids, aldehydes, ketones, phenols, furans, alcohols, sugars, and esters, where acids were the dominant compounds, with a relative content of 37.05−42.77%.

## 1. Introduction

Regarded as a renewable natural composite material, wood has been widely used to produce construction materials, flooring, furniture, and interior finishing materials because of its versatile properties, e.g., favorable strength-to-weight ratio, ease of shaping with tools, as well as beautiful grain and color [1,2]. However, the outdoor utilization of wood is highly limited by its strong hygroscopicity and low durability. Thermal modification (TM) is an ecological and low-cost pretreatment method to improve the dimensional stability and decay resistance of wood without using any toxic chemicals [3]. It is normally performed at between 160 to 260 °C in a vacuum, nitrogen, air, or oil environments [4,5,6]. 

During the last decade, thermal modifications of wood have been extensively studied and applied commercially [7,8,9,10,11,12,13]. Bruno et al. found that dimensional stability in the radial and tangential directions increased by 88% and 96% at 200 °C, respectively [14]. Bal et al. studied the effect of temperature (120, 150, and 180 °C) and the duration (4, 6, and 8 h) of thermal modification on the mechanical properties (e.g., MOE, MOR, compression strength, and impact bending) of Eucalyptus grandis; it was found that these properties decreased with an increase of TM temperature and duration. However, the influence of temperature was more remarkable than that of duration [15]. Candelier et al. showed that the bending strength of TM wood decreased by about 45%, and the elastic modulus by about 12% at 230 °C [16]. Lin et al. investigated the variation of chemical structure and composition of wood at different TM temperatures (200, 210, 220 and 230 °C), and found that the hygroscopic hydroxyl and oxygen element was remarkably removed by dehydration reactions, resulting in great improvements in dimensional stability [17]. However, a systematic investigation on the effect of TM temperature and duration on the chemical structure and the physical-mechanical properties (e.g., dimensional stability, color, and surface functional group) of oak (*Quercus alba* L.) has not been reported.

During thermal modification processes, a certain amount of volatile organic compounds (VOCs) will be released from the thermal degradation of wood, such as terpenes, aldehydes, acids, and alcohols. The emission of exhaust organic gas containing high concentrations of VOCs might result in serious atmospheric contamination, which could negatively affect human health. Traditionally, the identification of VOCs comprised two steps: VOCs were collected by condensation or extraction techniques, and then analyzed using a gas chromatography/mass spectrometer (GC/MS) [18,19,20]. Liu et al. extracted the VOCs adsorbed in activated carbon using methylene chloride and analyzed them using GC/MS. The result showed that butanedioic acid, bis (2-methylpropyl) ester was the dominant component in the volatiles of a pipe thermal modification process, accounting a content of 40.67% [21]. Kačík et al. extracted the terpenes adsorbed in sawdust using hexane and analyzed them using GC/MS; it was found that recent fir wood contained approximately 60 times more terpenes than older wood (186 vs. 3.1 mg/kg) [22]. Manninena et al. compared the content and components of VOCs in air-dried and heat-treated pine wood, and found that the former released about 8 times more VOCs than the latter [23]. Hyttinen et al. also found that the levels of VOCs released from heat treated wood were much lower than those from air-dried wood [24]. However, according to the literature, the traditional detection of VOCs is a complex process, requiring a long-period of experimentation and showing poor repeatability. Therefore, developing a quick and simple online detection method is essential for gaining a better understanding of the properties of VOCs. 

A thermogravimetric analyzer coupled with Fourier transform infrared spectrometry (TGA-FTIR) and a pyrolizer coupled with a gas chromatography/mass spectrometer (Py-GC/MS) were traditionally used to online investigate the components of pyrolysis volatiles of lignocellulosic biomasses [25,26,27]. TGA-FTIR analysis makes it possible to investigate weight loss characteristics during biomass thermal degradation processes, as well as to identify the evolved gas components in real time, especially for the small molecular weight gas components (H_2_O, CO_2_, CO, and CH_4_) [28,29]. Py-GC/MS was developed for the further qualitative and quantitative real-time analysis of each organic component in the volatiles, providing the advantages of rapid analyses, high sensitivity, and effective identification of complex organic compounds released from the wood thermal modification processes [30,31]. Until now, these two instruments have been extensively employed to analyze the components of pyrolysis volatiles of different lignocellulosic biomasses [29,32,33], or their three pseudo components, cellulose [34], hemicellulose [35,36], and lignin [26,31,37]. However, the application of these two instruments in the research field of wood TM has not been reported.

In this study, the thermal modification of white oak (*Quercus alba* L.) was carried out at different temperatures (160, 180 and 200 °C) and holding times (3, 6, 9 h). Then, the relevance between the evolution of chemical structure (e.g., elementary composition, surface functional group, and crystallinity) and the physical-mechanical properties (e.g., dimensional stability, MOE, MOR, color, and contact angle) were systematically investigated using a Universal Testing Machine, Elementary Analyzer, Colorimeter, FTIR, and XRD. Furthermore, the release characteristics of VOCs was online detected by TGA-FTIR and Py-GC/MS.

## 2. Materials and Methods 

### 2.1. Materials 

**Figure 1:**
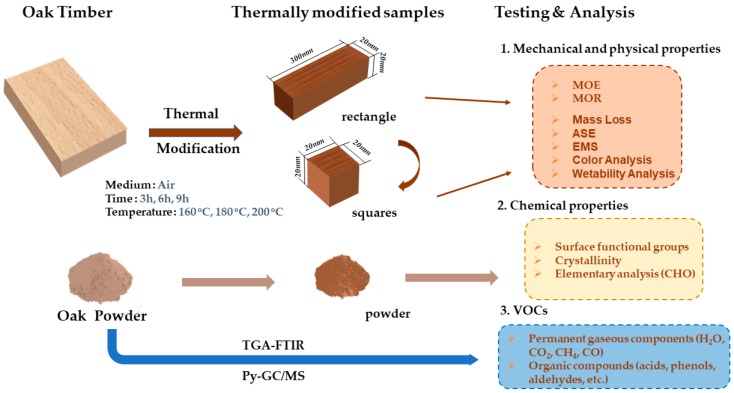
Flow diagram of wood thermal modification experiment.

White Oak (*Quercus alba* L.) was used for the thermal modification experiment; samples were purchased from Treessun Flooring Co. Ltd., Huzhou City, Zhejiang Province, China. The Oak was first cut into different sizes, depending on the different test methods, such as a dimension of 300 mm × 20 mm × 20 mm (*L* × *W* × *H*) for the test of bending strength, 30 mm × 20 mm × 20 mm (*L* × *W* × *H*) for the test of shrinkage, and powder with particle sizes between 220 to 280 meshes for the test of the release of VOCs. Before the thermal modification experiment, the sample was packaged inside sealed plastic bags and stored in a dryer at room temperature. The flow diagram of the wood thermal modification experiment is shown in Figure 1. The statistical analysis (standard deviation and *p*-value) of all physical and mechanical properties of TM wood are listed in Tables S1–S7 (Supplementary Materials).

### 2.2. Thermal Modification Experiment

The thermal modification experiment of oak was carried out in an oven (WFO-710, Shanghai Ailang instrument Co., LTD, Shanghai, China) with an air atmosphere. The designed temperatures for heat treatment were 160, 180, and 200 °C and the durations were 3, 6, and 9 h. The thermally-modified (TM) samples at different temperatures and durations were labeled as TM-xxx-y, where “xxx” represented the temperature of the heat treatment and “y” the duration. Fox example, TM-180-3 represented a sample which was treated at 180 °C for 3 h. All thermal modification experiments were repeated at least 3 times.

#### 2.2.1. Mechanical and Physical Properties

The mass loss (ML) of wood after thermal modification was determined by Equation (1), where *m*_0_ is the initial mass of the untreated sample, and *m*_1_ is the mass after thermal modification.

ML (%) = 100 × (*m*_0_ − *m*_1_)/*m*_0_ (1)

Prior to the test of mechanism properties, the raw and thermally-modified samples were conditioned at 20 °C and 65 % relative humidity for the necessary time to stabilize the mass of the samples. The equilibrium moisture content (EMC) and anti-swelling efficiency (ASE) was tested according to the national standard of GB/T 1931-2009 and GB/T 1934.2-2009, respectively. The modulus of elasticity (MOE) and modulus of rupture (MOR) were tested according to the national standards, i.e., GB/T 1936.2-1991 and GB/T 1936.1-2009, respectively.

#### 2.2.2. Color Analysis

The variations of color of the TM samples were measured by a colorimeter (DC−P3, Beijing Xingguang Color Measuring Instrument Co., Ltd., Beijing, China). In this instrument, a D65 light source, a 10° visual field, and a sensor head with 6 mm diameter were employed. Then, the color parameters of control and TM samples, namely L* (lightness coordinate), a* (red and green coordinates), and b* (yellow and blue coordinates) were recorded. In order to ensure the accuracy of the results, the color was measured on three specific places on each sample. Finally, the total color differences (ΔE*) were calculated according to Equation (2):

(2) $$\Delta E^{*}=\sqrt{\Delta L^{*2}+\Delta a^{*2}+\Delta b^{*2}},$$

where ΔL*, Δa*, and Δb* are the differences of parameters before and after TM.

#### 2.2.3. Chemical Properties

The ultimate analysis (C, H, and O) of the raw and TM samples was performed using an elemental analyzer (Vario EL III, Elementary, Germany). The chemical functional groups of the raw and TM samples were tested by Fourier transform infrared spectrometry (Nicolet 6700, Thermo Fisher Scientific, Massachusetts, USA). The mass ratio of KBr to bio-char was 100. The resolution and spectral region of the recorded FTIR spectra were 4 cm^−1^ and 4000–400 cm^−1^, respectively, and the spectrum scan time was set at 8 s intervals.

The crystallographic structure of the raw and TM samples was tested using an X-ray diffractometer (XRD 6000, Shimadzu, Kyoto, Japan) with Cu radiation at 40 kV and 30 mA. Scans were performed at a speed of 0.5° min^−1^ over an angle (θ) range of 5° to 40°. The crystallinity index (CrI) was calculated using Equation (3) according to Segal et al. [38], where C_r_I is the crystallinity index, *I*_002_ represents the intensity of the 200 crystalline peaks, and *I*_am_ represents the intensity of the diffraction of the amorphous part.

CrI (%) = 100 × (*I*_002_ − *I*_am_)/ *I*_002_ (3)

The contact angle was measured using optical contact angle measuring and contour analysis systems (OCA 200, Data Physics Instruments GmbH, Filderstadt, Germany), and through the disposition of a distilled water droplet (5 μL) in three distinct points of a tangential section of the wood samples. The data was recorded after 20 s of the droplet contacting the sample surface.

### 2.3. Online Analysis of VOCs by Using TGA-FTIR and Py-GC/MS

#### 2.3.1. TGA-FTIR Analysis

A thermogravimetric analyzer coupled with Fourier transform infrared spectrometry (TGA-FTIR) allowed us to investigate the weight loss characteristics during wood thermal modification processes, as well as to identify the evolution of the gas components in real time, especially for small molecular weight gaseous components (H_2_O, CO_2_, CO, and CH_4_). The instrument models of the TGA and FTIR were TGA-8000 and Frontier, respectively, both of which were made by PerkinElmer Co., Ltd, Waltham, MA, USA. The settled thermal modification temperatures were 160, 180, and 200 °C, with a fixed heating rate of 10 °C min^−1^ and a holding time of 30 min. In order to enhance the intensity of the infrared characteristic absorption peaks of the permanent gas components, 35 mg wood powder was used in each experiment. The carried gas was high-purity nitrogen (99.999%) with a flow rate of 40 mL min^−1^. The resolution and spectral region of the FTIR were 4 cm^−1^ and 4000–400 cm^−1^ respectively, and the spectrum scan time was set at 8 s intervals. More detailed information on the experiment may be found in our previous publications [27,39].

According to the Lambert-Beer law, the intensity of absorbance of a characteristic infrared absorbance band is linearly dependent on the concentration of the evolved gas component. In order to normalize FTIR data for comparison, first, the same initial mass (35 mg) of samples was used for TGA-FTIR analysis. Then, the experimental parameters in the TGA-FTIR analysis were fixed, e.g., heating rate (10 °C min^−1^), holding time (30 min), flow rate of carrier gas (40 mL min^−1^), spectrum scan time (8 s). The only variable was the thermal modification temperature (160, 180, and 200 °C), to investigate the effect of TM temperature on the properties of VOCs.

#### 2.3.2. Py–GC/MS Analysis

Py–GC/MS was used for the qualitative and semi-quantitative analyses of the organic compounds during wood thermal modification processes. The organic compounds released from such processes were on-line analyzed using a pyrolizer (5200, Chemical Data Systems Analytical, Oxford, Pennsylvania, USA) coupled with a gas chromatography/mass spectrometer (7890B-5977B, Agilent Technology, Palo Alto, California, USA). First, 0.5 mg of wood powder was put into a quartz filler tube and then heated to the target torrefaction temperatures of 160, 180, and 200 °C at a heating rate of 10 °C ms^−1^, and maintained for 20 s. The GC oven was first heated to 40 °C for 3 min, then raised to 290 °C (10 °C min^−1^) and maintained at that temperature for 3 min. The organic components were identified according to the NIST library and the literature. Other experimental information may be found in references [30,32,40].

## 3. Results and Discussion

### 3.1. Mass Loss 

**Figure 2:**
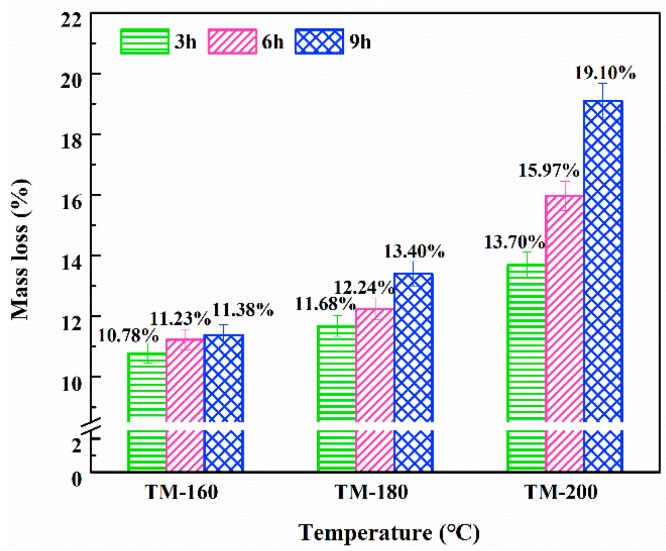
Effects of thermal treatment on the mass loss of wood.

The effect of TM on mass loss (ML) is shown in Figure 2. The results showed that higher temperatures and longer durations led to an increase of ML, ranging between 10.78% and 19.10%. In addition, temperature had a more remarkable influence on ML than duration. This result was confirmed by other researchers [7]. Srinivas et al. found that ML gradually increased from 3% to 18% with an increase of temperature and duration of TM from 210 °C and 2 h to 240 °C and 8 h, respectively [41]. Wang et al. also reported that ML reached its maximum value of 18.1% under the severest TM conditions, i.e., 190 °C and 6 h [42]. In the low temperature range (<160 °C) of TM, the mass loss was a consequence of the evaporation of free and hydroscopic water [17]. However, at higher temperatures (>160 °C), the mass loss was mainly due to the thermal degradation of hemicellulose [3]. This was caused by the fact that hemicellulose showed the lowest thermal stability among the three biomass pseudo components (cellulose, hemicellulose, and lignin) within a temperature range of 100–365 °C [28]. The mass loss in wood transfers into other small molecular weight components, such as CO, CO_2_, H_2_O, CH_4_, and VOCs, etc.

### 3.2. XRD Analysis

**Figure 3:**
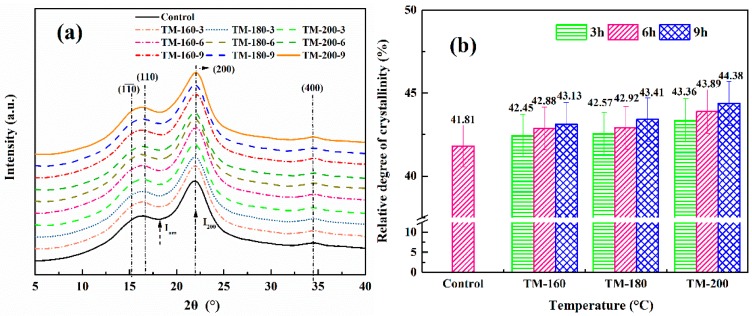
XRD analysis of the control and thermally-modified wood: (**a**) the XRD spectra; (**b**) the crystallinity index (CrI).

The XRD spectra of control and thermally-modified samples are shown in Figure 3a. Four characteristic diffraction peaks at 2θ of 15.2°, 16.5°, 22.2° and 34.6° were clearly observed, corresponding to the triclinic cellulose *I*_α_ ( $1\bar{10}$ and 110), monoclinic cellulose *I*_β_ (200), and the glucan chains of cellulose (400) [43]. The most remarkable variation was observed in the peak of monoclinic cellulose *I*_β_ (200); the intensity of this peak slightly increased with the increase of temperature and duration, indicating an increase in the crystallinity index (CrI) of cellulose. This result was caused by the thermal degradation of part of the hemicellulose and the rearrangement of cellulose molecules in the amorphous region [42,44]. As shown in Figure 3b, the value of CrI increased from 41.81% in the control sample to 44.38% of TM-200-9. Other references also reported the similar conclusions [5,45]. Okon et al. reported that CrI increased from 38.83% to 63.78% of the control sample from an oil heat treatment at 210 °C and 8 h [5]. Wang et al. also found that the CrI increased from 55.55% of the control sample to 63.33% after TM treatment at 190 °C and 6 h [42]. The decrease of the amorphous region in cellulose would result in a decrease of hydroxyl (–OH), leading to a significant reduction in the hygroscopicity of wood after TM [46].

### 3.3. FTIR Analysis

**Figure 4:**
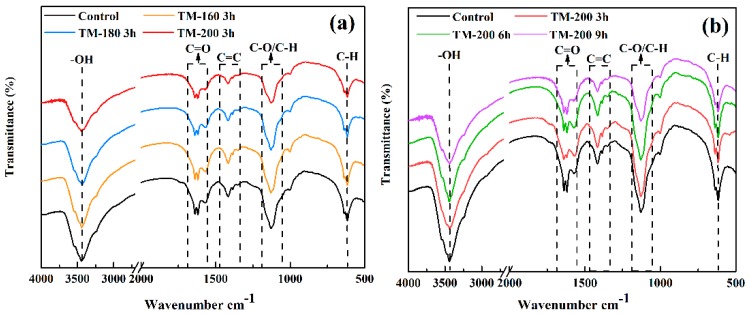
FTIR analysis of the control and thermally-modified wood.

The evolution of the surface functional groups of wood at different TM temperatures and durations is exhibited in Figure 4. Five characteristic absorbance bands may be clearly observed in the IR spectra of control and TM wood. The first band is the stretching vibration of hydroxyl (−OH) at the wavenumber of 3460 cm^−1^ [47,48]. The band at 1706 cm^−1^ is ascribed to the stretching vibration of C=O, which is mainly derived from the carbonyl (−C=O) and carboxyl functional groups (−COOH) [49]. The characteristic absorbance peak between 1680−1440 cm^−1^ is the stretching vibration of a benzene ring skeleton (C=C) from lignin [47]. The absorbance peak at 1190−950 cm^−1^ is attributed to the stretching vibration of C−O and C–H derived from aliphatic –CH_3_ or phenolic–OH bonds [17,50]. The band at 592 cm^−1^ is mainly due to aliphatic −CH_2_ and alkanes −CH_3_ [39,51].

Figure 4a shows the effect of TM temperature on the surface functional groups of wood. Overall, the absorbance intensity of the five characteristic adsorption bands decreased as the torrefaction temperature increased from 160 to 200 °C. The decrease of the intensity of −OH indicated that a series of deacetylation reactions had occurred; this resulted in the formation of H_2_O during TM process, and in a remarkable increase in hydrophobicity [3,50]. The decrease of the intensity of C=O was likely due to decarboxylation and decarbonylation reactions within the structures of cellulose and hemicellulose. The slight decrease of the intensity of C=C or the benzene ring skeleton indicated the thermal degradation of lignin. The decrease of C−O and C−H indicated the degradation of methyl and hydroxyl groups. The band of the aliphatic −CH_2_ and alkanes −CH_3_ exhibited a reduced absorbance intensity, indicating that the aliphatic regions of cellulose and hemicellulose were degraded. Figure 4b shows the effect of TM duration on the surface functional groups of wood. Overall, longer TM durations also resulted in the decrease of the absorbance intensity of the five adsorption bands. FTIR was also employed by Cademartori et al. and Sikora et al. to investigate the effect of TM on the content of surface functional groups of wood. Their results also confirmed that the intensities of several characteristic absorbance peaks decreased with the increase of TM temperature and duration [4,52].

### 3.4. Ultimate Analysis

The ultimate analysis (C, H, and O element) of the control and TM wood is shown in Table 1. The content of H and O was slightly decreased with an increase of the severity of TM. Fox example, the content of H and O decreased from 6.01% and 47.26% of TM-160-3 to 5.92% and 45.84% of TM-200-9, respectively. The reduction of H and O strongly supports the conclusion that a series of dihydroxylation (−OH) reactions occurred during the TM process, resulting in an increase in dimensional stability [3,53]. However, the content of C increased from 46.74% of TM-160-3 to 48.14% of TM-200-9 [54]. Boonstra et al. also found that the content of C slightly increased from 49.6%, in non-treated samples, to 50.6% in TM samples at 180 °C [3].

### 3.5. Contact Angles 

**Figure 5:**
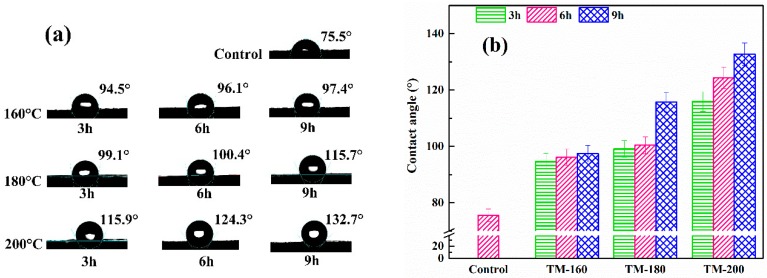
Contact angle in the tangential section of the control and thermally-modified woods.

The wettability of TM samples was evaluated by the contact angle, where a large contact angle corresponded to greater hydrophobicity and better dimensional stability [55]. Figure 5a shows the pictures of the contact angle testing process. Higher TM temperatures and longer durations would result in round-shaped water droplets on the surface; otherwise, flatter shapes were observed, since the liquid was rapidly absorbed, leading to small contact angles [56].

Figure 5b shows the effect of the severity of thermal modification on the contact angle in the tangential section of samples. For the control sample, the contact angle was only 67.63°. However, with the increase of the severity of TM, the contact angles gradually increased to 132.7° for the sample of TM-200-9, indicating that TM was an effective method to improve the hydrophobicity of wood. This result was caused by the dehydration reaction of carbohydrates during thermal modification, reducing the number of hydrophilic groups and restraining the accessibility of free hydroxyl groups to water [51,53]. A similar trend was confirmed by other researchers. Bakar et al. found that the the contact angle of red oak increased from 68° to 143° of the control sample under TM conditions of 190 °C and 8 h [56]. Lee et al. reported that the contact angle of bamboo increased from 53.2° to 116.8° of control sample at TM conditions of 210 °C and 4 h [57]. After treatment at higher temperatures, the contact angles were all higher than 90°, suggesting that TM pretreatment is highly beneficial for surface hydrophobicity.

### 3.6. EMC and ASE

**Figure 6:**
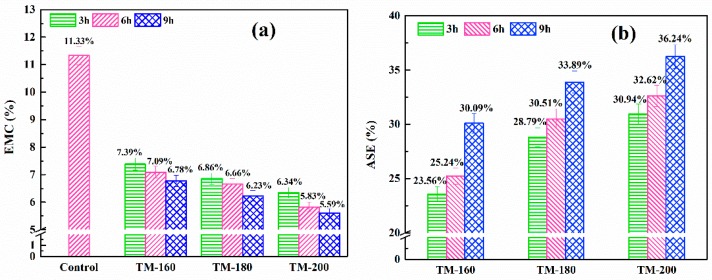
Effect of thermal modification on the EMC (**a**) and ASE (**b**).

Figure 6a shows the effect of TM on the equilibrium moisture content (EMC). Compared to the control sample, the EMC of thermally-modified samples was markedly decreased. Meanwhile, increasing the severity of TM would result in a lower value of EMC, decreasing from 7.39% of TM-160-3 to 5.59% of TM-200-9. The decrease of wettability was caused by the elimination of hydroxyl (–OH) linked on hemicellulose and the reduction of the amorphous region of cellulose, which reduces hydrogen bond interactions between the hemicellulose/cellulose and the water from the humid atmosphere [2,3,58].

As shown in Figure 6b, the anti-shrink efficiency (ASE) was also improved with an increase in the severity of TM. The ASE was increased from 23.56% of TM-160-3 to 36.24% of TM-200-9. This result was also confirmed by Ayrilmis et al. and Gonzálezpeña et al [48,59]. Wang et al. found that the EMC of the control sample decreased from 11.3% to 6.6% in TM-190-6 [42]. The ASE of TM samples is positively correlated with TM severity, and a maximum value of 56% was observed in TM-190-6. Although the EMC and ASE of wood are dominantly affected by TM temperature and duration, the influence factor of wood species cannot be ignored [59]. For example, eucalyptus has higher swelling and lower dimensional stability in nature [59]. In conclusion, TM is an effective pretreatment method to improve the dimensional stability of wood.

### 3.7. MOR and MOE

**Figure 7:**
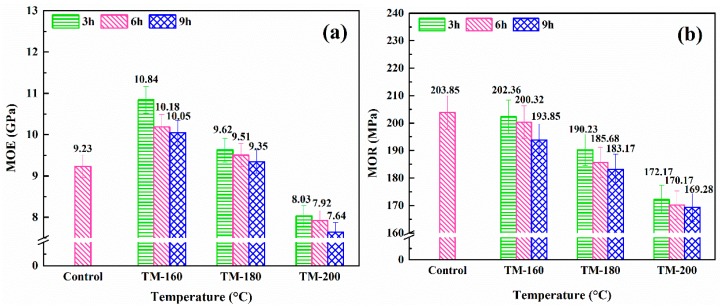
Effect of thermal modification on MOE (**a**) and MOR (**b**).

The Modulus of Elasticity (MOE) and Modulus of Rupture (MOR) are two important mechanical properties of wood. Figure 7 shows the effect of TM temperature and duration on MOE and MOR. As show in Figure 7b, the MOR was gradually decreased with an increase of temperature and duration. Fox example, the MOR was gradually decreased from 203.85 MPa of the control sample to 169.28 MPa of TM-200-9. The decrease of MOR was mainly due to the acceleration of the thermal degradation of hemicellulose at higher temperatures and longer durations [41,60,61]. This result was confirmed by other researches [59,60]. Ayrilmis et al. reported that after TM at 180 °C, the MOR and MOE of eucalyptus wood fibers decreased by 5–19% and 7–22%, respectively [59]. The decrease of MOR was highly related to the thermal degradation of cellulose and hemicellulose.

However, the MOE firstly increased from 9.23 GPa of the control sample to 10.84 GPa of TM-160-9, and then gradually decreased to 7.64 GPa of TM-200-9 (shown in Figure 7a). Similar results were obtained in studies conducted by Guo et al. and Yildiz et al. [62,63]. Guo et al. studied the effect of TM on the mechanical properties of white poplar (Populous tomentosa.). The results showed that the MOE firstly increased by 13% after TM at 200 °C for 1 h, before decreasing by 9% after TM at 250 °C for 5 h [62]. The increase of MOE at 160 °C and 180 °C was mainly due to the increase of the crystallization of cellulose and condensation of lignin via cross-linking reactions with furfural produced from the thermal degradation of hemicellulose [15,42,64].

### 3.8. Color Analysis

**Figure 8:**
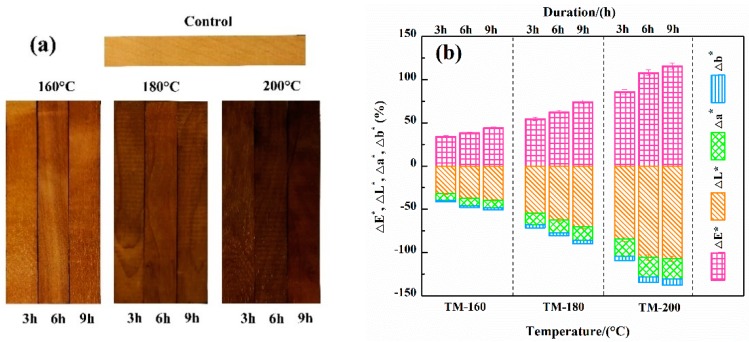
Surface color (**a**) and color parameters (**b**) of the control and thermally-modified woods.

Figure 8a shows the surface color of the control and TM woods. The results showed that TM could induce remarkable color variations on the wood surface; higher temperatures and durations were associated with darker color. The variations of color parameters (ΔL*, Δa*, Δb*, ΔE*) before and after thermal modification are shown in Figure 8b. The ΔL* (lightness coordinate), Δa* (red/green coordinate), and Δb* (yellow /blue coordinate) were negative values derived from the difference values before and after thermal modification. Researchers reported that higher difference values indicated large color variation [4,57]. The absolute values of ΔL*, Δa*, Δb*, and ΔE* all increased with an increase of the severity of TM. The increase of the absolute value of ΔL* from −32.20 of TM-160-3 to −107.50 of TM-200-9 indicated a reduction of lightness. A similar trend was observed on Δa*, which increased from −7.23 of TM-160-3 to −23.25 of TM-200-9, resulting in the surface taking on a reddish color. The increase of the absolute value of Δb* from −1.85 of TM-160-3 to −6.86 of TM-200-9 resulted in the surface taking on a yellow color. The increase of total color differences (ΔE*) indicated the variation trend of surface color towards darker tones, which was in accordance with the color variation shown in Figure 8a.

The variation of surface color was caused by the increase of chromophores formed in the TM process. Firstly, the acetic acid formed by the deacetylation of hemicellulose will act as a catalyst to promote the oxidation and dehydration reactions of lignin or carbohydrates to form new chromophores, particularly, carbonyl and carboxyl groups [41,51]. Then, the acetic acid will also promote the substitution reaction of free hydroxyl groups to form ether bonds, and polycondensation of phenolic hydroxyl groups to form oxidation products such as conjugated aromatic ketone and quinones, resulting in a progressively darker wood color [2,60,65]. Bekhta et al. reported that changes in the color of spruce wood, i.e., becoming darker and redder, were due to the enrichment of phenolics on the wood surface. The Δb* of wood after TM at 150 °C was 6–7 times that of the control sample [66]. In addition, besides the TM temperature and duration, Sundqvist et al. reported that the content of extractives (e.g. phenols, ketones and quinones) also has a strong influence on the color [41,65].

### 3.9. TG-FTIR Analysis

**Figure 9:**
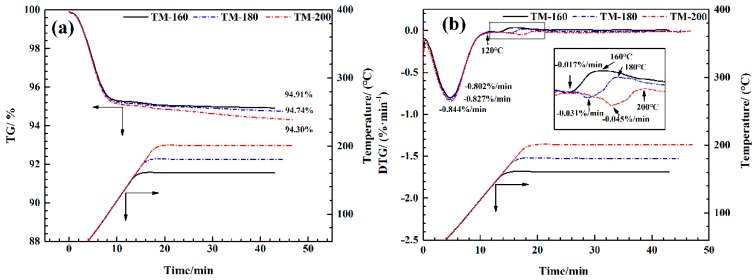
TG (**a**) and DTG (**b**) curves of thermal modification process the wood.

TGA-FTIR analysis makes it possible to investigate weight loss characteristics during biomass thermal degradation processes, as well as to identify the evolution of gas components in real time, especially for the small molecular weight bio-gas components (H_2_O, CO_2_, CO, and CH_4_). Figure 9 shows the thermogravimetry (TG) and derivative thermogravimetry (DTG) curves of a wood thermal modification process with a heating rate of 10 °C min^−1^. Based on the TG curves shown in Figure 9a, the residual mass decreased from 94.91% to 94.30% as the thermal modification temperature increased from 160 to 200 °C. This result indicates that higher thermal modification temperatures lead to higher mass loss in wood.

As shown in Figure 9b, two distinct mass loss peaks were observed. The first indicates the dehydration stage (30–120 °C), resulting from the evaluation of free and bound water in wood [17,39]. With the increase of TM temperature from 160 to 200 °C, the mass loss rate at the first peak was slightly increased, i.e., 0.802% to 0.844%/min. The second peak was formed by the thermal degradation of hemicellulose and cellulose in the wood [50,67]. Higher thermal modification temperatures resulted in a wider temperature range of mass loss and in higher mass loss rates. For example, the temperature range of this peak increased from 120–160 °C to 120–200 °C, and the mass loss rate increased from 0.017% to 0.045%/min. The mass lost in this stage mainly transferred into small molecular weight gaseous components (CO, CO_2_, CH_4_ and H_2_O) and VOCs [3,50,52].

### 3.10. 3D−FTIR Analysis

**Figure 10:**
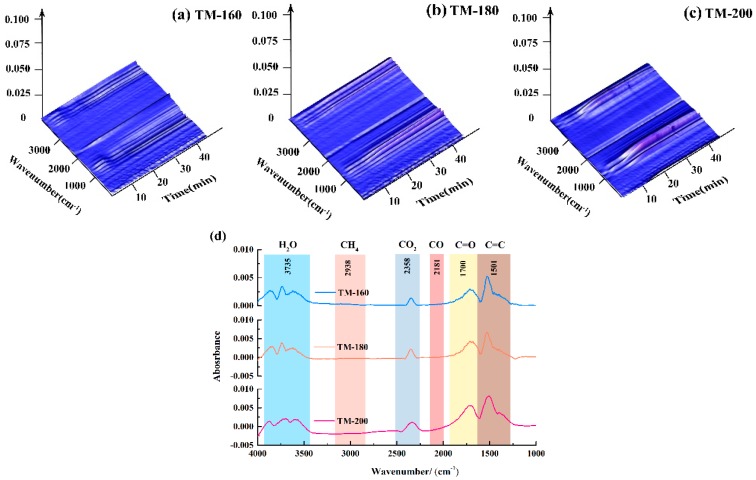
3D-FTIR analysis of wood thermal modification at different temperatures: (**a**) 3D-FTIR of TM-160; (**b**) 3D-FTIR of TM-180; (**c**) 3D-FTIR of TM-200; (**d**) 2D-FTIR at the points of maximum weight loss from the three thermal modification samples.

Figure 10a–c shows the 3D-FTIR spectra of wood thermal degradation at three different temperatures (160, 180, and 200 °C). The intensity of absorbance significantly increased as the thermal modification temperature increased from 160 to 200 °C, indicating that higher temperatures promote the formation of evolved gas components. In order to identify the components of the evolved gas, a 2D-FTIR diagram with the wavenumber as the x-axis and the adsorbance intensity as the y-axis is presented (Figure 10d). Some permanent gaseous components could be easily identified according to their characteristic infrared absorbance bands, such as those of H_2_O at 3735 cm^−1^, CH_4_ at 2938 cm^−1^, CO_2_ at 2358 cm^−1^, and CO at 2181 cm^−1^ [49,50].

**Figure 11:**
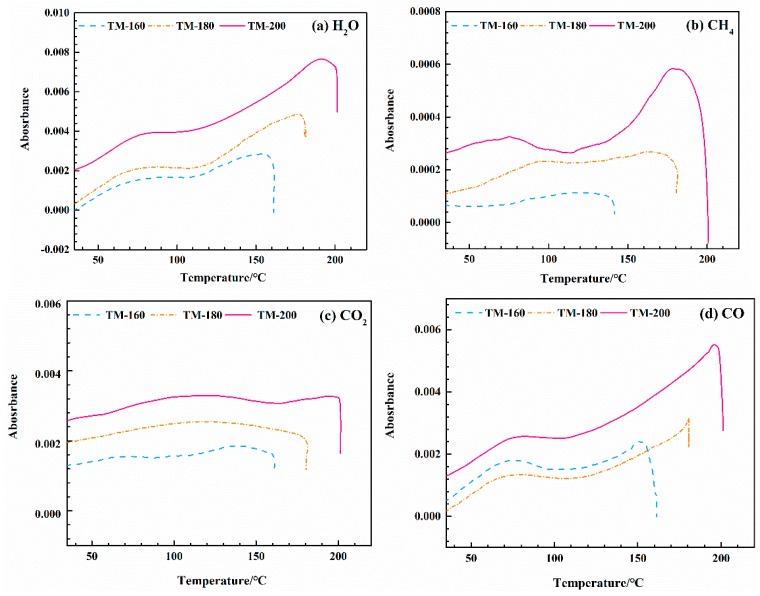
Evolution of evolved gas components during wood thermal modification at different temperatures: (**a**) H_2_O; (**b**) CH_4_; (**c**) CO_2_; and (**d**) CO.

After the identification of components at characteristic infrared absorbance bands, the evolution of each detected gas component (H_2_O, CH_4_, CO_2_, and CO) was determined; see Figure 11. According to the Lambert-Beer law, the intensity of the absorbance of a characteristic infrared absorbance band is linearly dependent on the concentration of the evolved gas components [68,69]. As shown in Figure 10, the intensities of the characteristic infrared absorbance bands of four gas components gradually increased with the increase of thermal modification temperature. Among the four gas components, H_2_O had the highest absorbance intensity, i.e., 0.008, at a thermal modification temperature of 200 °C, followed by CH_4_, i.e., 0.006, CO, 0.0058, and CO_2_, about 0.0038.

The release of H_2_O with increasing TM temperature can be divided into two stages. At temperatures lower than 100 °C, the release is due to the evaporation of free water [17], while at higher temperatures, it is due to the breakage of hydroxyl groups linked to the glycosyl in hemicellulose [25]. The release of CO_2_ can be mainly attributed by the decarbonylation and decarboxylation reactions of C=O and –COOH groups linked to the glucuronic acid units of hemicellulose [39,49,50]. The formation of CO was found to be mainly due to the cracking of carboxyl groups during ring-opening reactions of the glycosyl unit in hemicellulose [4,48,49]. The formation of CH_4_ was also mainly due to breakages of methyl (–CH_3_) and methylene (–CH_2_–) linked to the lateral chain of the glycosyl unit in hemicellulose [39,63]. The acceleration of the thermal degradation of hemicellulose resulted in an increased release of these four gas components at higher thermal modification temperatures.

### 3.11. VOCs Analysis by PY-GC/MS 

**Figure 12:**
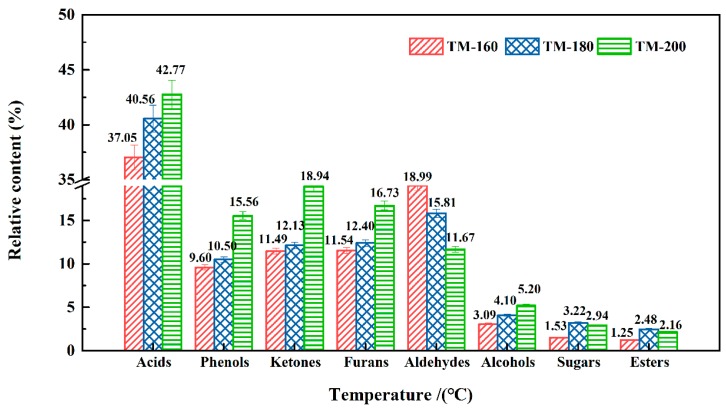
The relative contents of components in VOCs released from different thermal modification temperatures.

The VOCs released from wood TM were online detected by Py-GC/MS. The compounds and their relative contents are listed in Table 2. According to the characteristic functional groups, the VOCs may be divided into eight groups, namely acids (37.05−42.77%), aldehydes (11.67−18.99%), ketones (11.49−18.94%), phenols (9.6−15.56%), furans (11.54−16.67%), alcohols (3.09−5.2%), sugars (1.53−3.22%), and esters (1.25−2.16%). The effect of thermal modification temperature on the relative contents of these groups is shown in Figure 12. Several publications have reported that monoterpenes (e.g. α-pinene, β-pinene, camphene, limonene, and β-phellandrene) are common compounds in VOCs derived from the TM of softwoods, such as Pine and Fir [20,22,23,70]. However, these compounds were not detected in the TM process of hardwood (white oak). The difference might be caused by the different chemical compounds in the extracts of soft- and hard- wood.

Among the eight groups’ chemicals, acids presented the highest relative content, i.e., 37.05−42.77%. The formation of acetic acid was mainly due to the thermal degradation of hemicellulose, which can occur via two pathways [35]. Firstly, acetic acid can be formed by the elimination of O–acetyl groups linked to xylan side chains at the C_2_ position; secondly, it can occur by the ring-opening reaction of the 4–O–methylglucuronic acid unit after the breakage of carbonyl and O–methyl groups. Sundman et al. and Hyttinen et al. also found that acids were the dominant compounds in VOCs. Sundman et al. also found that the maximum emission of acids was 2800 μg/(m^2^·h) [19]. Hyttinen et al. reported that acetic acid reached its maximum emission rate, i.e., 170 μg/(m^2^·h) on the 28th day over a five-week testing period [24].

The relative content of acids gradually increased with an increase of thermal modification temperature. This result indicates that more hemicellulose was degraded at higher temperatures. It is worth noting that about 50% of the acids was composed of long-chain fatty acids, such as heptadecanoic acid (10.2−12.43%) and eicosanoic acid (15.08−15.7%). This result indicates that lower thermal degradation temperature promotes the formation of long-chain fatty acids.

The relative contents of phenols increased from 9.6% to 15.56% at higher TM temperatures. Phenols were produced by the thermal degradation of lignin [26,31]. As shown in Table 2, trans-isoeugenol and (E)-2,6-dimethoxy-4-(prop-1-en-1-yl) phenol are the two dominant components in phenols, with relative abundances of 2.23−2.26% and 3.74−5.77%, respectively. These two components could be directly obtained from the cleavage of β–O–4 linkages in the lignin [25]. 

With the increase of the thermal modification temperature, the relative contents of ketones and furans increased from 11.49% and 11.54 % to 15.94% and 16.73%, respectively, while the content of aldehydes decreased from 18.99% to 11.67%. The increase in furans and ketones was mainly attributed to ring-opening and depolymerization reactions of the basic structural units of glucan in cellulose, and glycosyl in hemicellulose [25,71]. Under lower TM temperatures, hemicellulose thermal degradation was the dominant process. However, as the TM temperature increased to over 200 °C, cellulose started to degrade, and therefore, higher TM temperatures promoted the formation of furans and ketones. Hyttinen et al. investigated the effect of heat treatment on the release content of VOCs, and found that furans were the major degradation products of hemicellulose, and that the content of furans gradually increased from 7 μg/(m^2^·h) on the second day to 37 μg/(m^2^·h) on the 28th day [24].

## 4. Conclusions

The connection between the evolution of both chemical structure and physical-mechanical properties during wood TM process, as well as the release characteristics of VOCs, were systematically investigated. The results indicated that the dimensional stability (e.g. anti-shrink efficiency, contact angle, equilibrium moisture content) improved markedly due to the reduction of hydrophilic hydroxyl (–OH). However, the mechanical properties (MOE and MOR) decreased after thermal modification due to the thermal degradation of hemicellulose and cellulose. Based on the TGA-FTIR analysis, the small molecular gaseous components were composed of H_2_O, CH_4_, CO_2_, and CO, where H_2_O was the dominant component with the highest absorbance intensity, i.e., 0.008 at 200 °C. Based on a Py-GC/MS analysis, the VOCs were mainly composed of acids, aldehydes, ketones, phenols, furans, alcohols, sugars, and esters, where acids were the dominant compounds, with relative contents of 37.05−42.77%.

## Figures and Tables

**Table 1:** Ultimate analysis of the control and thermally-modified wood.

Element Content/(wt.%)	Control and Thermally-Modified Wood									
	Control	TM-160-3	TM-160-6	TM-160-9	TM-180-3	TM-180-6	TM-180-9	TM-200-3	TM-200-6	TM-200-9
C	45.83	46.74	46.89	46.95	47.07	47.68	47.62	47.62	47.93	48.14
H	6.46	6.01	5.99	5.93	6.04	6.04	5.95	6.00	5.97	5.92
O	47.60	47.26	47.10	47.00	46.99	46.16	46.34	46.26	45.95	45.84

**Table 2:** Compounds and their relative contents in VOCs released from wood thermal modification at different temperatures.

Category	RT (min)	Compounds	Relative Content (Peak Area%)		
			TM-160	TM-180	TM-200
Acids	4.44	Acetic acid	1.53	3.32	5.51
	7.91	2-Hydroxy-6-methyl-3-cyclohexen-1-carboxylic acid	1.47	1.39	1.29
	9.09	Z-3-Methyl-2-hexenoic acid	0.91	0.56	0.78
	9.43	1,2-Dimethylcyclopropanecarboxylic acid	0.23	0.20	0.17
	9.79	(E)-3-Methyl-4-decenoic acid	0.31	0.21	0.16
	11.87	2-Hydroxy-6-methyl-3-cyclohexen-1-carboxylic acid	0.21	0.32	0.47
	12.50	(E)-3-Hexenoic acid	0.28	0.75	1.02
	12.92	3-Ethyl-3-methyl-pentanedioic acid	0.16	0.25	0.37
	16.25	Dodecanoic acid	1.27	1.10	/
	17.67	7-Methoxybenzofuran-2-carboxylic acid	0.88	0.71	0.79
	17.97	3,5-Dimethoxy-4-hydroxyphenylacetic acid	0.76	0.33	0.70
	18.49	Tetradecanoic acid	1.06	1.23	0.39
	19.53	3,5-Dimethoxy-4-hydroxyphenylacetic acid	0.00	0.44	0.31
	20.35	n-Hexadecanoic acid	0.00	0.30	0.33
	20.60	Heptadecanoic acid	10.2	11.32	12.43
	21.50	(Z,Z)-9,12-Octadecadienoic acid	0.16	0.26	/
	22.19	Oleic Acid	0.69	1.36	1.41
	22.23	Octadecanoic acid	1.23	1.43	1.32
	22.50	Eicosanoic acid	15.70	15.08	15.32
	Total		37.05	40.56	42.77
Phenols	13.11	2-Methoxy-4-vinylphenol	1.33	0.90	1.07
	13.62	2,6-Dimethoxy-phenol	0.29	0.33	0.37
	13.70	Eugenol	0.40	0.35	0.28
	14.91	trans-Isoeugenol	2.26	2.23	2.24
	16.78	2,6-Dimethoxy-4-(2-propenyl)-phenol	0.47	0.43	0.63
	17.90	(E)-2,6-Dimethoxy-4-(prop-1-en-1-yl) phenol	3.74	4.18	5.77
	18.70	Desaspidinol	0.61	0.71	1.34
	19.98	5-(3-Hydroxypropyl)-2,3-dimethoxyphenol	0.34	0.31	0.50
	27.14	3,5-bis(1,1-Dimethylethyl)-1,2-benzenediol	0.00	0.00	0.31
	31.97	2,6-bis(1,1-Dimethylethyl)-1,4-benzenediol	0.16	1.06	3.05
	Total		9.60	10.50	15.56
Aldehydes	14.28	Vanillin	3.13	2.33	0.59
	15.28	4-(t-Butyl)benzaldehyde	0.57	0.38	0.24
	17.48	4-Hydroxy-3,5-dimethoxy-benzaldehyde	3.71	2.97	1.83
	18.31	4-Hydroxy-2-methoxycinnamaldehyde	5.8	5.26	4.02
	20.86	3,5-Dimethoxy-4-hydroxycinnamaldehyde	5.78	4.87	4.99
	Total		18.99	15.81	11.67
Ketones	8.38	2,4-Hexanedione	7.50	8.18	15.71
	15.96	1-(4-Hydroxy-3-methoxyphenyl)-2-propanone	0.38	0.36	0.38
	16.33	3′,5′-Dimethoxyacetophenone	1.27	1.73	1.88
	16.63	1-(2-Hydroxy-4-methoxyphenyl)propan-1-one	1.98	1.56	0.34
	19.34	1-[2-(5-hydroxy-1,1-dimethylhexyl)-3-methyl-2-cyclopropen-1-yl]-ethanone	0.36	0.30	0.28
	24.41	3-Tridecanoyl-3-cyclohexen-4-ol-1-one	0.00	0.00	0.35
	Total		11.49	12.13	18.94
Furans	5.39	2(5H)-Furanone	0.00	0.00	0.40
	6.18	Furfural	8.45	9.34	13.41
	11.99	5,6-Dihydro-6-pentyl-2H-pyran-2-one,	0.68	0.76	0.89
	12.23	6-Ethoxy-3,6-dihydro-3-hydroxy-2H-pyran-2-methanol	0.28	0.34	0.45
	13.24	5-Butyldihydro-4-methyl-2(3H)-furanone	0.38	0.25	0.14
	17.76	5-(1-Hexynyl)-furan-2-carboxylic acid	0.00	0.20	0.22
	19.26	5-tert-Butyl-2-(4-tert-butylphenoxymethyl)-furane-3-carboxylic acid	1.75	1.51	1.22
	Total		11.54	12.40	16.73
Alcohols	15.06	α-Ethyl-4-methoxy-benzenemethanol	0.76	0.22	0.29
	16.33	(2α,3α,4α)- 2-(3,4-dimethoxyphenyl)-3,4-dihydro-6-methyl-2H-1-benzopyran-3,4-diol	1.27	0.73	1.88
	17.35	4-Hydroxy-3-methoxy-benzenepropanol	0.67	0.64	0.62
	27.786	(all-E)-(±)- 2,6,10,15,19,23-hexamethyl-1,6,10,14,18,22-tetracosahexaen-3-ol	0.39	2.51	2.41
	Total		3.09	4.10	5.20
Sugars	11.16	2-Deoxy-D-galactose	0.91	0.66	0.44
	13.98	D-Allose	0.62	0.7	0.75
	15.45	1,6-Anhydro-β-D-glucopyranose	0.00	1.86	1.75
		Total	1.53	3.22	2.94
Esters	10.14	Heptamethylene diacetate	0.38	0.42	0.55
	12.23	Carbonic acid-but-2-yn-1-yl undecyl ester	0.28	0.38	0.45
	22.11	Heptadecanoic acid-16-methyl-methyl ester	0.38	0.29	0.11
	23.73	Octadecanoic acid-2-hydroxy-1,3-propanediyl ester	0.00	0.74	0.21
	25.68	Diisooctyl phthalate	0.00	0.30	0.51
	25.96	Cholesteryl formate	0.21	0.35	0.33
	Total		1.25	2.48	2.16

## Supplementary Materials

The following are available online at https://www.mdpi.com/2073-4360/11/7/1145/s1, Table S1: The P-level analysis of data from the physical-mechanical properties, Table S2: Mean and standard deviation values of mass loss of thermal modified wood, Table S3: Mean and standard deviation values of crystallinity index (CrI) of the control and thermal modified wood, Table S4: Mean and standard deviation values of ultimate analysis of the control and thermal modified wood, Table S5: Mean and standard deviation values of the EMC and ASE of the control and thermal modified wood, Table S6: Mean and standard deviation values for the MOE and MOR of the control and thermal modified wood, Table S7: Mean and standard deviation values of the surface color of the control and thermal modified wood.
