# Supplementary material for: New Perspective on Wood Thermal Modification: Relevance between the Evolution of Chemical Structure and Physical-Mechanical Properties, and Online Analysis of Release of VOCs
Source: Polymers (Basel). 2019 Jul 4;11(7):1145. doi: 10.3390/polym11071145 (PMC6680412; doi:10.3390/polym11071145)
Supplement: Supplementary file 1 [file polymers-11-01145-s001.pdf]

New Perspective on Wood Thermal Modification: Relevance between the Evolution of Chemical Structure and Physical-Mechanical Properties, and Online Analysis of Release of VOCs

Jiajia Xu [1,†], Yu Zhang [1,†], Yunfang Shen [2], Cong Li [1], Yanwei Wang [3], Zhongqing Ma [1,*] and Weisheng Sun [1,*]

1 School of Engineering, Zhejiang Provincial Collaborative Innovation Center for Bamboo Resources and High-Efficiency Utilization, Zhejiang A & F University, Hangzhou, Zhejiang 311300, China
2 Zhejiang Shenghua Yunfeng Greeneo Co. Ltd., Huzhou, Zhejiang 313220, China
3 Treessun Flooring Co. Ltd., Huzhou, Zhejiang 313009, China
* Correspondence: mazq@zafu.edu.cn (Z.M.); sunweisheng@zafu.edu.cn (W. S.); Tel.: 86-571-6110-0905 (Z.M. & W.S.)
† These authors were contributed equally to this manuscript.

Table S1. The P-level analysis of data from the physical-mechanical properties

Properties	Type	Sig.	p-level
ML (%)	TM-160-3/TM-200-9	0.000	<0.001
C (%)	TM-160-3/TM-200-9	0.019	<0.005
H (%)	TM-160-3/TM-200-9	0.001	<0.005
O (%)	TM-160-3/TM-200-9	0.003	<0.005
CrI	TM-160-3/TM-200-9	0.000	<0.001
EMC (%)	TM-160-3/TM-200-9	0.000	<0.001
ASE (%)	TM-160-3/TM-200-9	0.000	<0.001
MOR (MPa)	TM-160-3/TM-200-9	0.001	<0.001
MOE (GPa)	TM-160-3/TM-200-9	0.000	<0.001
ΔE*	TM-160-3/TM-200-9	0.000	<0.001
ΔL*	TM-160-3/TM-200-9	0.000	<0.001
Δa*	TM-160-3/TM-200-9	0.000	<0.001
Δb*	TM-160-3/TM-200-9	0.000	<0.001

Table S2. Mean and standard deviation values of mass loss of thermal modified wood.

TM	ML (%)	
	Mean	Std. dev.
TM-160-3	10.78	0.15
TM-160-6	11.23	0.13
TM-160-9	11.38	0.14
TM-180-3	11.68	0.19
TM-180-6	12.24	0.18
TM-180-9	13.40	0.18
TM-200-3	13.70	0.17
TM-200-6	15.97	0.22
TM-200-9	19.10	0.34

Table S3. Mean and standard deviation values of crystallinity index (CrI) of the control and thermal modified wood

TM	CrI(%)	
	**Mean**	**Std. dev.**
Control	41.81	0.35
TM-160-3	42.45	0.37
TM-160-6	42.88	0.39
TM-160-9	43.13	0.45
TM-180-3	42.57	0.23
TM-180-6	42.92	0.64
TM-180-9	43.41	0.23
TM-200-3	43.36	0.40
TM-200-6	43.89	0.24
TM-200-9	44.38	0.47

Table S4. Mean and standard deviation values of ultimate analysis of the control and thermal modified wood.

TM	C /(wt.%)		H /(wt.%)		O /(wt.%)	
	**Mean**	**Std. dev.**	**Mean**	**Std. dev.**	**Mean**	**Std. dev.**
Control	45.83	0.47	6.46	0.32	47.60	0.67
TM-160-3	46.74	0.45	6.01	0.22	47.26	0.50
TM-160-6	46.89	0.56	5.99	0.16	47.10	0.67
TM-160-9	46.95	0.63	5.93	0.08	47.00	0.72
TM-180-3	47.07	0.48	6.04	0.04	46.99	0.72
TM-180-6	47.68	0.10	6.04	0.02	46.16	0.15
TM-180-9	47.62	0.97	5.95	0.01	46.34	0.26
TM-200-3	47.62	0.53	6.00	0.04	46.26	0.35
TM-200-6	47.93	0.42	5.97	0.03	45.95	0.26
TM-200-9	48.14	0.18	5.92	0.24	45.84	0.72

Table S5. Mean and standard deviation values of the EMC and ASE of the control and thermal modified wood.

TM	EMC (%)		ASE (%)	
	**Mean**	**Std. dev.**	**Mean**	**Std. dev.**
Control	11.33	0.87	/	/
TM-160-3	7.39	0.26	23.56	2.12
TM-160-6	7.09	0.39	25.24	2.00
TM-160-9	6.78	0.36	30.01	3.43
TM-180-3	6.86	0.42	28.79	2.83
TM-180-6	6.66	0.27	30.31	3.71
TM-180-9	6.23	0.20	33.90	2.12
TM-200-3	6.34	0.26	30.95	3.48
TM-200-6	5.83	0.36	32.62	3.61
TM-200-9	5.59	0.37	36.24	4.26

Table S6. Mean and standard deviation values for the MOE and MOR of the control and thermal modified wood.

TM	MOE (GPa)		MOR (MPa)	
	Mean	Std. dev.	Mean	Std. dev.
Control	9.23	0.89	203.85	7.01
TM-160-3	10.84	1.21	202.36	10.71
TM-160-6	10.18	0.94	200.32	11.79
TM-160-9	10.05	0.63	193.85	10.20
TM-180-3	9.62	0.96	190.23	8.65
TM-180-6	9.51	0.57	185.68	9.47
TM-180-9	9.35	0.82	183.17	8.54
TM-200-3	8.03	0.54	172.17	6.54
TM-200-6	7.92	0.53	170.17	6.80
TM-200-9	7.64	0.54	169.28	6.83

Table S7. Mean and standard deviation values of the surface color of the control and thermal modified wood.

Samples	ΔL*		Δa*		Δb*		ΔE*	
	Mean	Std. dev.	Mean	Std. dev.	Mean	Std. dev.	Mean	Std. dev.
TM-160-3	34.38	1.71	−34.38	1.97	−7.25	0.89	−1.85	0.38
TM-160-6	38.43	1.72	−37.55	2.56	−8.24	0.53	−2.21	0.40
TM-160-9	44.04	2.41	−39.47	2.98	−8.52	1.07	−2.65	0.44
TM-180-3	54.51	2.71	−54.57	2.13	−13.45	1.15	−3.56	0.51
TM-180-6	62.69	2.36	−62.58	2.09	−14.47	0.64	−3.72	0.54
TM-180-9	74.19	3.79	−69.98	3.58	−15.73	0.58	−4.25	0.64
TM-200-3	86.09	4.96	−84.73	4.53	−19.70	0.95	−4.68	0.65
TM-200-6	108.13	4.53	−105.87	4.87	−22.66	1.09	−6.48	0.85
TM-200-9	115.79	4.11	−107.51	5.11	−23.25	1.14	−6.86	0.68
